# Effect of implantable cardiac monitors on preventing stroke: A systematic review and meta-analysis of randomized clinical trials

**DOI:** 10.1371/journal.pone.0287318

**Published:** 2023-07-20

**Authors:** Jialing He, Yaxin Jiang, Yangchun Xiao, Pengfei Hao, Tiangui Li, Liyuan Peng, Yuning Feng, Xin Cheng, Haidong Deng, Peng Wang, Weelic Chong, Yang Hai, Lvlin Chen, Chao You, Lu Jia, Fang Fang, Shui Yu, Yu Zhang

**Affiliations:** 1 Evidence-Based Medicine Center, Affiliated Hospital of Chengdu University, Chengdu, Sichuan, China; 2 Department of Out-patient, West China Hospital, Sichuan University, Chengdu, Sichuan, China; 3 Department of Neurosurgery, Affiliated Hospital of Chengdu University, Chengdu, Sichuan, China; 4 Department of Neurosurgery, Shanxi Provincial People’s Hospital, Taiyuan, Shanxi, China; 5 Department of Neurosurgery, Longquan Hospital, Chengdu, Sichuan, China; 6 Department of Critical Care Medicine, Affiliated Hospital of Chengdu University, Chengdu, Sichuan, China; 7 Department of orthopedics, Affiliated Hospital of Chengdu University, Chengdu, Sichuan, China; 8 Department of Neurosurgery, West China Hospital, Sichuan University, Chengdu, Sichuan, China; 9 Department of Medical Oncology, Thomas Jefferson University, Philadelphia, PA, United States of America; 10 Sidney Kimmel Medical College, Thomas Jefferson University, Philadelphia, PA, United States of America; Hualien Tzu Chi Hospital Buddhist Tzu Chi Medical Foundation, TAIWAN

## Abstract

**Background and aim:**

Implantable cardiac monitors (ICM) can facilitate the detection of asymptomatic atrial fibrillation episodes. We performed a systematic review and meta-analysis to investigate whether ICM can prevent stroke in patients with prior stroke and risk factors for stroke.

**Methods:**

This study included randomized controlled trials comparing ICM with conventional (non-ICM) external cardiac monitoring in patients with prior stroke and risk factors for stroke. We searched Medline, Embase, and CENTRAL from inception until January 5, 2022, without language restriction. Quantitative pooling of the data was undertaken using a random-effects model. The primary outcome was ischemic stroke at the longest follow-up.

**Results:**

Four trials comprising 7237 patients were included. ICM was significantly associated with decreased risk of ischemic stroke (RR 0.76; 95% CI, 0.59–0.97; moderate-quality evidence) in patients with prior stroke and risk factors for stroke. ICM was associated with higher detection of atrial fibrillation (RR 4.21, 95% CI 2.26–7.85) and use of oral anticoagulants (RR 2.29, 95% CI 2.07–2.55).

**Conclusions:**

ICM results in a significantly lower risk of ischemic stroke than conventional (non-ICM) external cardiac monitoring in patients with prior stroke and risk factors for stroke. Due to the clinical heterogeneity of study population and limited related studies, more trials were needed to furtherly explore the topic in patients with prior stroke or high risk of stroke.

## Introduction

Stroke is a leading cause of mortality and disability globally [1]. Approximately one-third of all ischemic strokes are judged as occurring as a complication of atrial fibrillation [2]. This risk might be underestimated because it did not account for the effect of undiagnosed atrial fibrillation (AF) which is likely a significant contribution to cryptogenic stroke. Thus, the early detection of AF should logically be beneficial in stroke prevention with guideline-recommended oral anticoagulation treatment [3].

Implantable cardiac monitors (ICM), a prolonged monitoring device, represent a sensitive method for detecting infrequent episodes of AF and are minimally invasive and well-tolerated by most patients [4]. Current guidelines recommend that prolonged rhythm monitoring for AF is reasonable within six months of the index event for patients with cryptogenic stroke [5, 6]. For individuals who had a high risk of stroke, guidelines also recommend systematic or intense screening for AF [7–9]. These recommendations are based on evidence that evaluation with monitoring devices improves the detection of AF. To date, however, the effect of ICM on stroke prevention, though reasonable, is currently unproven. Previous individual randomized controlled trials (RCTs) have generally reported results suggestive of a preventive effect of ICM on stroke but failed to show statistical significance of this association. Thus, we combined the results of high-quality RCTs to investigate whether ICM can prevent stroke in patients with prior stroke and/or risk factors for stroke.

## Methods

We used methods recommended by the Cochrane Collaboration to conduct the meta-analysis. The methods of reporting study followed the Preferred Reporting Items for Systematic Reviews and Meta-Analyses (PRISMA) 2020 statement [10]. Given that this is a meta-analysis of published data, institutional review committee approval was not required. The protocol was registered on the PROSPERO (Register number CRD42022301705).

### Eligibility criteria

We included trials that met each of the following PICOS criteria:

1. Population: adult patients (≥18 years) with ischemic stroke or transient ischemic attack and patients with a high risk of ischemic stroke (e.g., aged >70 years, patients with hypertension, diabetes or heart failure, patients with dementia or those living in a long-term care home, patients with acute ischemic stroke and patients with an intracardiac thrombus)
2. Intervention: ICM
3. Comparison intervention: Any form of non-ICM external rhythm monitoring performed in the control group (e.g., 12-lead electrocardiogram, Holter, event recorders, 30-d external loop recorder and mobile cardiac telemetry)
4. Outcome: Ischemic stroke event (either recurrent or new).
5. Study design: RCTs

### Information sources and search strategy

We searched Medline, EMBASE, and Cochrane Central Register of Controlled Trials without any language restrictions. The last electronic search was performed on January 5, 2022. We also searched the references lists of previous reviews, meta-analyses, and RCTs. The details of the search strategy are seen in S1 Table.

### Study selection

Two reviewers (JH and PH) independently screened titles and abstracts and the full text of potentially relevant studies. Disagreements between the two reviewers were resolved by discussion and adjudication by a third reviewer (YZ).

### Data collection process

Two reviewers (JH and PH) performed data extraction independently and extracted data using a standardized electronic form. Disagreements between the two reviewers were resolved by discussion and adjudication by a third reviewer (YZ). Study authors were contacted when suitable data were not available. Unpublished data were included after receiving a response and additional data from study investigators.

### Outcomes

The primary outcome was ischemic stroke at the longest follow-up. A composite outcome of ischemic stroke and TIA was used to compute the pooled analysis on ischemic stroke unless actual ischemic stroke were reported or were obtained from study authors. Secondary outcomes were AF, use of oral anticoagulants, major bleeding, hemorrhagic stroke, and all-cause mortality.

### Assessment of risk of bias

Two reviewers (JH and PH) independently applied the Cochrane Risk of Bias tool for randomized controlled trials [11]. Disagreements between the two reviewers were resolved by discussion and adjudication by a third reviewer (YZ). The risk of bias for each domain was assessed as high, low, or unknown.

### Confidence of evidence

We used the Grading of Recommendation, Assessment, Development, and Evaluation (GRADE) approach to generate an absolute and relative risk of the outcomes [12]. GRADE guidance used the domains of study design limitations, inconsistency, indirectness, publication bias, and imprecision in results.

### Data synthesis

We used RevMan version 5.4 (Cochrane Collaboration) and R software version 4.1.2 for all meta-analyses, with a P-value < 0.05 denoting statistical significance. Analyses for all outcomes were done on an intention-to-treat basis. For dichotomous outcomes, we conducted random-effects meta-analysis using the Mantel-Haenszel (M-H) method with 95% CI. We assessed statistical heterogeneity using the Chi^2^ test and the I^2^ test. Random-effects model was applied for all meta-analyses. We assessed evidence of publication bias across studies using funnel plots if ten or more studies were in a meta-analysis [11].

### Subgroup analysis

We conducted subgroup analyses based on variables (1) the incidence of AF in control groups (≥30% vs. <30%), (2) the type of ICM, and (3) type of patients (prior stroke vs high risk of stroke).

### Sensitivity analyses

Sensitivity analyses were conducted for the primary outcomes by (1) excluding trials published before 2015, (2) using fixed-effect models, (3) excluding trials with fewer than 400 patients, (3) excluding trials with high risk or unknown risk of bias in the different domains, (4) excluding trials that reported a composite outcome of ischemic stroke.

## Results

### Study selection

**Fig 1:**
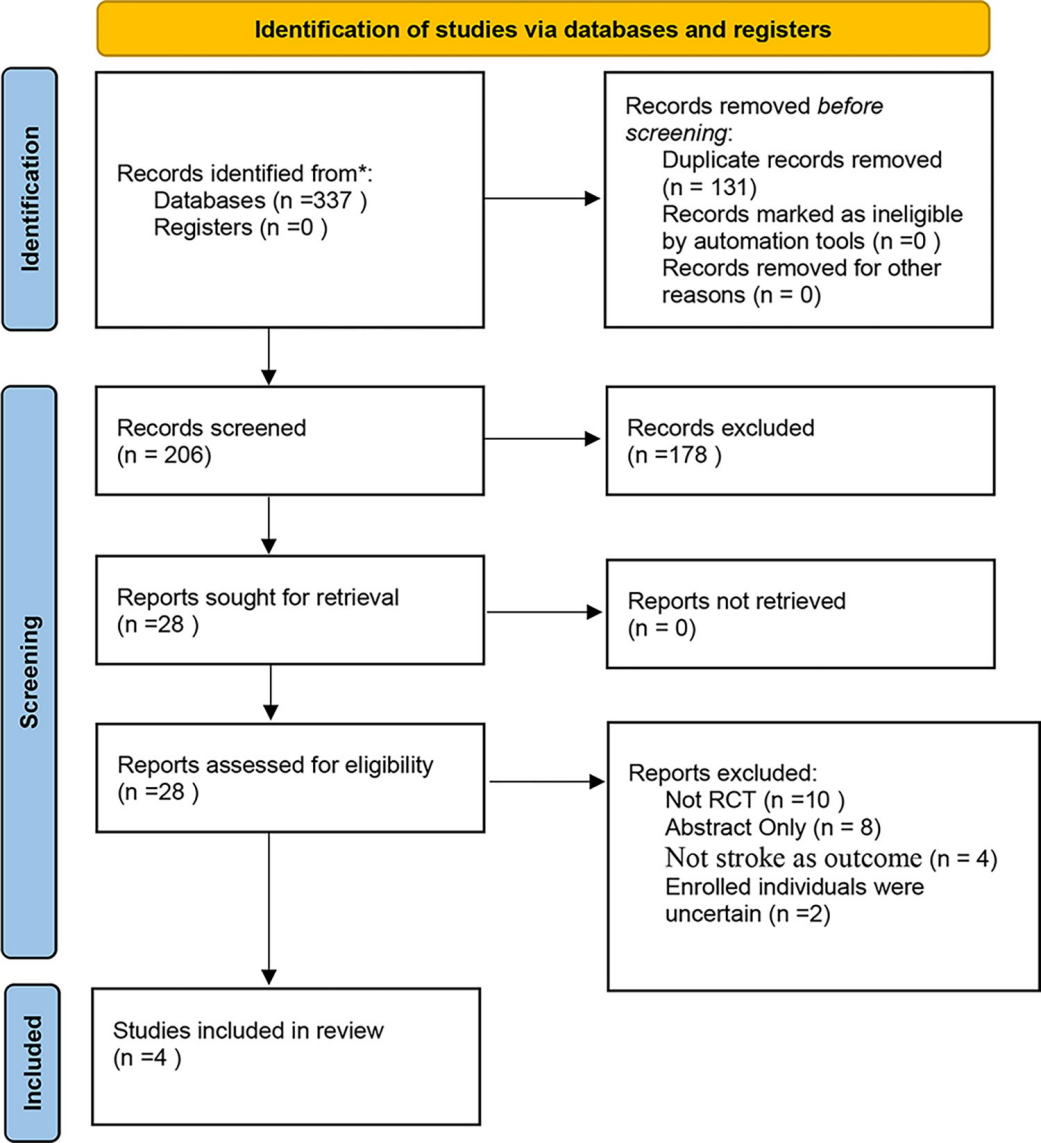


Fig 1 shows the PRISMA flow diagram of the meta-analysis. We initially identified 337 potentially eligible publications. Ultimately, four trials were deemed eligible for this meta-analysis. The four trials were The Cryptogenic Stroke and Underlying AF (CRYSTAL AF) [13], The Stroke of Known Cause and Underlying Atrial Fibrillation (STROKE-AF) [14], the Post-Embolic Rhythm Detection with Implantable versus External Monitoring (PERDIEM) [15], and Atrial Fibrillation Detected by Continuous ECG Monitoring Using Implantable Loop Recorder to Prevent Stroke in High-risk Individuals (LOOP) [16].

### Study characteristics

The characteristics of the included trials and patients are demonstrated in Table 1. Three trials included patients with prior stroke, and one trial included patients with a high risk of stroke. All trials excluded patients with a prior history of AF. Mean age ranged from 61 to 68 years in the three trials of patients with prior stroke, and mean age was 74 in the trial of patients with a high risk of stroke. CHADS2/ CHA2DS2-VASc scores were similar across trials.

**Table 1:** Baseline patient characteristics and treatment parameters by treatment group among included randomized trials.

Trials	CRYSTAL AF	PERDIEM	STROKE-AF	LOOP
Year	2014	2021	2021	2021
No of site	55	3	33	4
Country	Europe, Canada, and the United States	Canada	the United States	Denmark.
No of patients	441	300	492	6004
Mean Age, y	ICM: 61.6	ICM: 65.5	ICM: 66.6	ICM: 74.7
	CG: 61.4	CG: 63.4	CG: 67.5	CG: 74.7
Women, %	ICM: 35.7	ICM: 40.7	ICM: 40	ICM: 47.2
	CG: 37.3	CG: 40.0	CG: 35.6	CG: 47.3
Eligible patients	≥ 40 years	≥18 years	≥60 years	70–90 years
	Stroke or TIA within 90 days	Stroke or TIA within 180 days	Stroke within 10 days	at least one additional stroke risk factor
Index event, %				
Stroke	91.0	NA	100	NA
TIA	11.2	NA	NA	NA
Comorbidities factors, %				
Hypertension	61.5	62.3	80.7	90.7
Diabetes mellitus	16.3	20	38	28.5
Heart failure	NA	2	10.4	4.4
Vascular disease	5.7	3	18.7	2.7
Hyperlipidemia	57.4	NA	NA	NA
Previous stroke	14.7	23.3	NA	17.6
CHADS2 score	3(2–3)	NA	NA	NA
CHA2DS2-VASc score*	NA	4 (3–5)	5(4–5)	4 (3–4)
Type of ICM	Medtronic REVEAL XT	Medtronic REVEAL LINQ	Medtronic REVEAL LINQ	Medtronic REVEAL LINQ
Control Group	ECG at scheduled and unscheduled visits	30-d external loop recorder	ECG, telemetry, Holter, or event recorders	ECG, annual interview
Primary Outcome	AF at 6 months	AF at 12 months	AF at 12 months	Combined endpoint^#^
Lost to follow-up, %	0.5	6.3	4.5	0
Follow-up for stroke, y	1	1	1	6

AF: atrial fibrillation; ICM: Implantable cardiac monitor; CG: Control group; ECG: electrocardiogram. *Values are presented as median (interquartile range)

# The combined endpoint of ischemic or hemorrhagic stroke, systemic embolism, bleeding leading to hospitalization, and all-cause death

### Risk of bias and quality of evidence

The overall risk of bias was moderate among studies (S1 and S2 Figs). The nature of the trial interventions precluded blinding of patients and their physicians; only one trial was not blinding of outcome assessment [15]. GRADE summary findings for all outcomes are shown in Table 2, and the quality of evidence for the primary outcome was moderate. This study did not assess the existence of possible publication bias because of the small number of included trials.

**Table 2:** Summary of findings and strength of evidence.

Outcome	NO. Of patients (Trials)	RR (95%CI)	Absolute effect estimates (per 1000)			Quality of the evidence
			Control	Intervention	Difference	
**Ischemic stroke**	7237(4)	0.76[0.59,0.97]	50	38	-12[–20, –1]	Moderate^†^
**Detection of AF**	7237(4)	4.21[2.26,7.85]	110	463	355[139,757]	High
**Oral anticoagulants**	7237(4)	2.29[2.07,2.55]	122	279	157[131,189]	High
**Major bleeding**	6304(2)	1.26[0.95,1.67]	34	43	9[–2,22]	Moderate*
**Hemorrhage stroke**	7237(4)	1.02[0.54,1.92]	7	7	0[–3,6]	Moderate*
**All-cause mortality**	6796(3)	0.89[0.59,1.34]	106	94	-12[–44,36]	Moderate*

CI: confidence interval; RR: risk ratio; AF: atrial fibrillation

^†^Inconsistency

* Imprecisions

### Ischemic stroke

**Fig 2:**
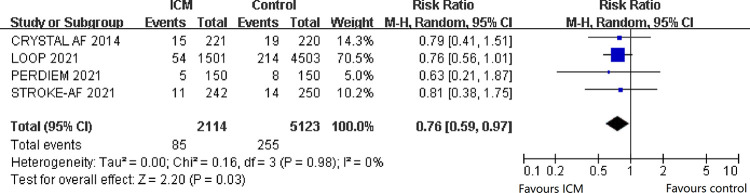


Fig 2 shows the primary outcome, ischemic stroke, which included four trials with 7237 patients. The PER DIEM Trial [15] and STROKE-AF Trial [14] reported ischemic stroke, and the CRYSTAL AF trial [13] reported a composite of ischemic stroke or TIA. The LOOP trial [16] did not report ischemic stroke in the article, but the corresponding author of the LOOP trial shared the data of ischemic stroke with us. Overall, the incidence of ischemic stroke was 4.0% for patients in the ICM arm and 5.0% in the comparison arm. The RR (0.76; 95% CI, 0.59–0.97; I^2^ = 0%) revealed an association between implantable cardiac monitors and a decreased risk of ischemic stroke.

The overall findings for ischemic stroke were similar when trials were excluded in sensitivity analyses, including trials reporting a composite of ischemic stroke or TIA, trials published earlier than 2015, trials with fewer than 400 patients, trials with non-low risk of bias, or pooling trials using fixed-effect models (S2 Table).

The subgroup analyses of the primary outcome did not show any interaction in variables, including the rate of AF in control groups, type of ICM, and type of patients (S3 Fig).

### Secondary outcomes

**Fig 3:**
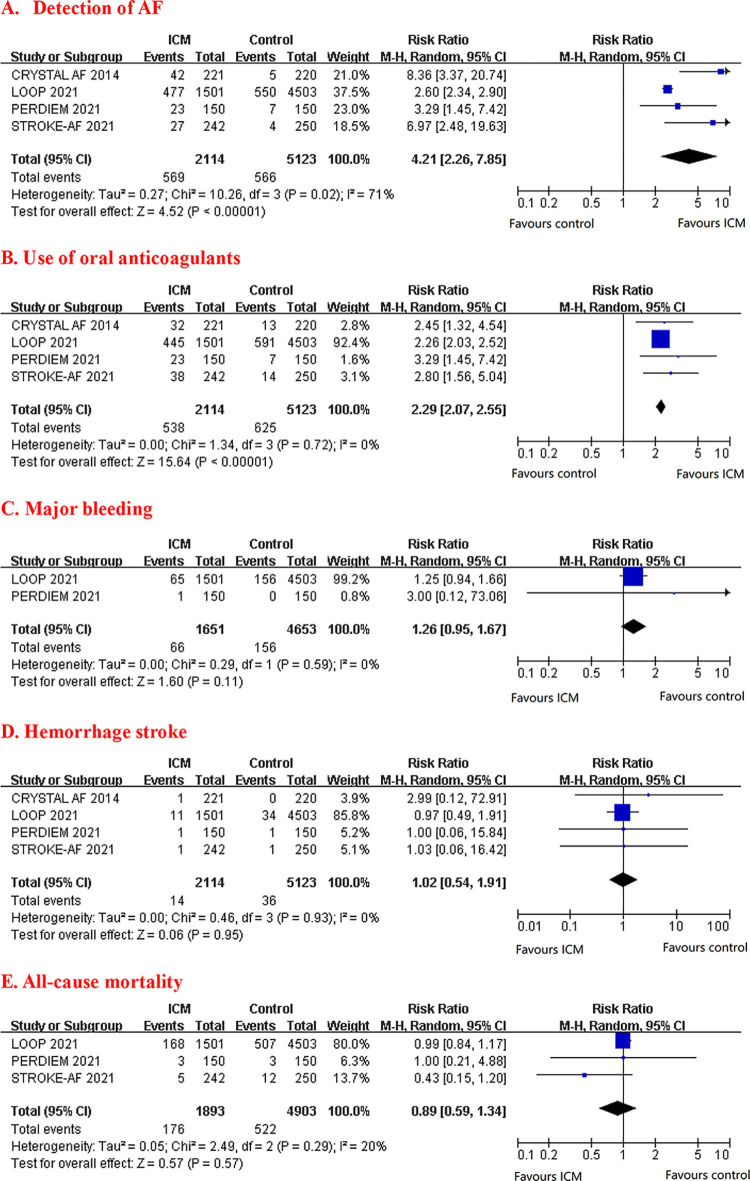


Results of secondary outcomes are detailed in Fig 3. ICM was associated with higher detection of AF (RR 4.21, 95% CI 2.26–7.85) and use of oral anticoagulants (RR 2.29, 95% CI 2.07–2.55). However, there was no significant difference between types of cardiac monitoring in major bleeding (RR 1.26, 95% CI 0.95–1.67), hemorrhage stroke (RR 1.02, 95%CI 0.54–1.91), and all-cause mortality (RR 0.89, 95% CI 0.59–1.34).

## Discussion

In this systematic meta-analysis of four RCTs with a total of 7237 patients, we found that compared to conventional (non-ICM) external cardiac monitoring, implantable cardiac monitoring detected significantly more AF events, in line with results from individual studies. Intriguingly, ICM decreased the relative risk for stroke by 24% compared with conventional (non-ICM) external cardiac monitoring in patients with prior stroke or risk factors for stroke.

Two systematic review and meta-analysis provide preliminary evidence from observational studies for a potential impact of prolonged cardiac monitoring on secondary stroke prevention but no solid evidence from RCT to support it [17, 18]. There are some differences between previous meta-analysis and our study. First, the included RCTs were not identical due to inconsistent intervention measures. The intervention measure of previous study was prolonged cardiac monitoring, including ICM and Holter, etc., but that of our study was only ICM. Second, our study population included patients with prior stroke and risk factors for stroke, but that of previous study only include patients with prior stroke. Third, our study did not consider observational cohort studies which previous study included, because observational cohort studies might have selection bias and heterogeneity, which might affect the reliability and accuracy of the results.

Previous meta-analysis found that ICM has shown high sensitivity and specificity for detecting AF [19–23]. The most important question with any intervention in clinical studies is whether it will impact clinically meaningful or ‘hard’ outcomes. The reasoning behind ICM trials is that a diagnosis of AF is required to initiate anticoagulant therapy after ischemic stroke, and this medication can prevent recurrent strokes. This hypothesis is reasonable but currently unproven. There are 4 RCTs of the use of ICM to prevent stroke in patients with prior stroke and risk factors for stroke. However, all four trials showed no significant differences in the outcomes of preventing stroke. The primary limitations of the first three trials were small numbers of events and relatively short follow-up because the primary outcome was AF detection but not the number of stroke events. The LOOP trial was designed to investigate whether ICM can prevent stroke in patients at high risk of stroke and anticipated a 35% decrease in the primary outcome–time to first stroke or systemic arterial embolism. Yet, the primary outcome occurred in 67 participants (4.5%) in the ICM group versus 251 (5.6%) in the control group (HR 0.80 [95% CI 0.61–1.05]). The overall risk reduction was only 20% and insufficient to reach significance. Thus, the study authors conclude that not all atrial fibrillation is worth screening for, and not all screen-detected atrial fibrillation merits anticoagulation. It is worth noting that in the ICM arm, 463 per 1000 people had AF, but only 279 per 1000 were prescribed oral anticoagulants, implying that in the trials, not all with AF were treated with oral anticoagulants, and thus decision-making for anticoagulants depends on factors other than AF. Those in the ICM arm were more likely to receive an oral anticoagulant compared to standard of care, with an absolute difference of 157 per 1000 patients (122 vs. 279 per 1000 in comparator and ICM, respectively), and whether this constitutes an over-prescription is still not well understood. There is a decreased incidence of stroke by 12 events per 1000 patients (50 per 1000 versus 38 per 1000 in the comparator and ICM arm, respectively) (Table 2). When the absolute increase in anticoagulation use and the absolute decrease in stroke events were considered, a number needed to treat of 13 was obtained.

This systematic review and meta-analysis has some key strengths. As far as we know, our study is the first meta-analysis to report that ICM (and therapy change associated with AF detection) can lead to the prevention of ischemic stroke. This study has a comprehensive search strategy, and we contacted the authors of all studies for any unpublished data on ischemic stroke. Our successful contact with authors [16] yielded important additional data to determine the effect of ICM on the prevention of stroke, which increased the precision and overall certainty of the effect estimates. Our study implemented a rigorous assessment of the quality of evidence, as well as relative and absolute risks, which are important for making decisions between ICM and conventional (non-ICM) external cardiac monitoring.

This study has some limitations. First, there was clinical heterogeneity in the meta-analysis. One trial enrolls patients with high risk of stroke (LOOP study) but has no stroke yet (primary prevention). The other three trials enrolled patients with a history of stroke (secondary prevention). The difference in population may be small. In the subgroup analysis based on the type of patients, both groups have the same RR (0.76), suggesting that the effect of ICM on both types of patients was similar. However, because the results were imprecise in both groups, more RCTs are needed to assess the effect of ICM in both populations. Second, the primary outcome is mainly driven by LOOP study due to its large study population. More studies are needed to furtherly validate our results. Third, all the included studies were not specifically designed to investigated the occurrence of stroke during follow-up and actual data on ischemic stroke occurrence were missing for CRYSTAL-AF (which reported a composite of ischemic stroke or TIA) and for LOOP Study (the corresponding authors shared unreported data with the authors). In sensitivity analysis, however, the meta-analysis results for ischemic stroke were still robust after excluding the CRYSTAL AF trial. Fourth, our statistical analysis might not be powered for important secondary outcomes (e.g., mortality) due to the limited sample size for these outcomes. Fifth, the limited number of included trials led to an insufficient ability to detect the presence of publication bias [11, 24]. Sixth, all included trials were performed in high-income countries, and ICM has a relatively high initial cost. Thus, findings in this study may not be generalizable or applicable to countries with emerging economies. Seventh, ischemic stroke reported in most included trials was the secondary outcome of the included trials. Methods of collecting data for this secondary outcome might have been conducted differently from the primary outcome in the trials.

Future studies should be done to determine the optimal duration for the ICM to detect AF (current studies ranged from 30 seconds to 120 seconds) and evidence that the use of OAC therapy in patients with subclinical AF (detected by ICM) demonstrates a reduction in stroke rates. Moreover, cost-effectiveness analyses should be pursued to ascertain the value of ICM-based strategies to reduce the incidence of stroke.

## Conclusions

ICM resulted in a significant (~24%) reduction in the risk of stroke in patients with prior stroke and risk factors for stroke compared with conventional (non-ICM) external cardiac monitoring. Due to the clinical heterogeneity of study population and limited related studies, more trials were needed to furtherly explore the topic in patients with prior stroke or high risk of stroke.

## Supporting information

S1 Checklist PRISMA 2020 checklist. (DOCX)

S1 Table Search strategy. (DOCX)

S2 Table Sensitivity analysis of meta-analysis. (DOCX)

S3 Table Excluded studies after full-text review. (DOCX)

S1 Fig Risk of bias summary. (DOCX)

S2 Fig Risk of bias graph. (DOCX)

S3 Fig Subgroup analysis of meta-analysis. (DOCX)
