# Supplementary material for: Effect of implantable cardiac monitors on preventing stroke: A systematic review and meta-analysis of randomized clinical trials
Source: PLoS One. 2023 Jul 20;18(7):e0287318. doi: 10.1371/journal.pone.0287318 (PMC10358888; doi:10.1371/journal.pone.0287318)
Supplement: S1 Table — (DOCX) [file pone.0287318.s002.docx]

**S1 Table. Search strategy**

Ovid MEDLINE(R) and Epub Ahead of Print, In-Process, In-Data-Review & Other Non-Indexed Citations, Daily and Versions(R)

1 Stroke.mp. or exp Stroke/ 355030

2 Cerebrovascular Accident?.mp. 7711

3 CVA?.mp. 4627

4 Apoplexy, Cerebrovascular.mp. 1

5 Brain Vascular Accident?.mp. 10

6 Vascular Accident?, Brain.mp. 2

7 Cerebrovascular Stroke?.mp. 386

8 Stroke?, Cerebrovascular.mp. 141

9 Apoplexy.mp. 3393

10 Cerebral Stroke?.mp. 1745

11 Stroke?, Cerebral.mp. 478

12 Acute Stroke?.mp. 17506

13 Stroke?, Acute.mp. 645

14 Cerebrovascular Accident?.mp. 7711

15 Acute Cerebrovascular Accident?.mp. 251

16 1 or 2 or 3 or 4 or 5 or 6 or 7 or 8 or 9 or 10 or 11 or 12 or 13 or 14 or 15 365314

17 implantable loop recorder.mp. 566

18 insertable cardiac monitor.mp. 132

19 cardiac implantable electronic devices.mp. 985

20 17 or 18 or 19 1656

21 ((randomized controlled trial or controlled clinical trial).pt. or randomized.ab. or placebo.ab. or drug therapy.fs. or randomly.ab. or trial.ab. or groups.ab.) not (animals not (humans and animals)).hw. 4558791

22 16 and 20 and 21 83

EBM Reviews - Cochrane Central Register of Controlled Trials

1 Stroke.mp. or exp Stroke/ 63745

2 Cerebrovascular Accident?.mp. 16195

3 CVA?.mp. 883

4 Apoplexy, Cerebrovascular.mp. 1

5 Brain Vascular Accident?.mp. 0

6 Vascular Accident?, Brain.mp. 1

7 Cerebrovascular Stroke?.mp. 38

8 Stroke?, Cerebrovascular.mp. 72

9 Apoplexy.mp. 351

10 Cerebral Stroke?.mp. 214

11 Stroke?, Cerebral.mp. 209

12 Acute Stroke?.mp. 4689

13 Stroke?, Acute.mp. 395

14 Cerebrovascular Accident?.mp. 16195

15 Acute Cerebrovascular Accident?.mp. 24

16 1 or 2 or 3 or 4 or 5 or 6 or 7 or 8 or 9 or 10 or 11 or 12 or 13 or 14 or 15 67932

17 implantable loop recorder.mp. 139

18 insertable cardiac monitor.mp. 40

19 cardiac implantable electronic devices.mp. 72

20 17 or 18 or 19 250

21 ((randomized controlled trial or controlled clinical trial).pt. or randomized.ab. or placebo.ab. or drug therapy.fs. or randomly.ab. or trial.ab. or groups.ab.) not (animals not (humans and animals)).hw. 1325291

22 16 and 20 and 21 58

Embase

1 Stroke.mp. or exp Stroke/ 565393

2 Cerebrovascular Accident?.mp. 243304

3 CVA?.mp. 9986

4 Apoplexy, Cerebrovascular.mp. 3

5 Brain Vascular Accident?.mp. 16

6 Vascular Accident?, Brain.mp. 4

7 Cerebrovascular Stroke?.mp. 636

8 Stroke?, Cerebrovascular.mp. 242

9 Apoplexy.mp. 3831

10 Cerebral Stroke?.mp. 2683

11 Stroke?, Cerebral.mp. 741

12 Acute Stroke?.mp. 32748

13 Stroke?, Acute.mp. 1542

14 Cerebrovascular Accident?.mp. 243304

15 Acute Cerebrovascular Accident?.mp. 480

16 1 or 2 or 3 or 4 or 5 or 6 or 7 or 8 or 9 or 10 or 11 or 12 or 13 or 14 or 15 575889

17 implantable loop recorder.mp. 1182

18 insertable cardiac monitor.mp. 275

19 cardiac implantable electronic devices.mp. 1557

20 17 or 18 or 19 2978

21 ((randomized controlled trial or controlled clinical trial).pt. or randomized.ab. or placebo.ab. or drug therapy.fs. or randomly.ab. or trial.ab. or groups.ab.) not (animals not (humans and animals)).hw. 7757817

22 16 and 20 and 21 196
