# Supplementary material for: Effect of implantable cardiac monitors on preventing stroke: A systematic review and meta-analysis of randomized clinical trials
Source: PLoS One. 2023 Jul 20;18(7):e0287318. doi: 10.1371/journal.pone.0287318 (PMC10358888; doi:10.1371/journal.pone.0287318)
Supplement: S2 Table — (DOCX) [file pone.0287318.s003.docx]

**S2 Table. Sensitivity analysis of meta-analysis**

|  | NO. patients(trials) | RR | 95%CI | I^2^, % |
| --- | --- | --- | --- | --- |
| All trials | 7237(4) | 0.76 | 0.59,0.97 | 0 |
| Excluding trials published before 2015 | 6796(3) | 0.75 | 0.58,0.98 | 0 |
| Using fixed-effect models | 7237(4) | 0.76 | 0.59,0.97 | 0 |
| Excluding trials with fewer than 400 patients | 6937(3) | 0.77 | 0.60,0.99 | 0 |
| Excluding trials that reported a composite outcome of ischemic stroke | 6937(3) | 0.77 | 0.60,0.99 | 0 |
| Excluding studies with high or unknown risk of bias in the different domains |  |  |  |  |
| Sequence generation | 7237(4) | 0.76 | 0.59,0.97 | 0 |
| Allocation concealment | 7237(4) | 0.76 | 0.59,0.97 | 0 |
| Blinding of patients and personnel | 0 | NA | NA | 0 |
| Blinding of outcome assessors | 6937(3) | 0.77 | 0.60,0.99 | 0 |
| Incomplete outcome data | 7237(4) | 0.76 | 0.59,0.97 | 0 |
| Selective reporting | 7237(4) | 0.76 | 0.59,0.97 | 0 |
| Other bias | 7237(4) | 0.76 | 0.59,0.97 | 0 |
