# Supplementary material for: Effect of implantable cardiac monitors on preventing stroke: A systematic review and meta-analysis of randomized clinical trials
Source: PLoS One. 2023 Jul 20;18(7):e0287318. doi: 10.1371/journal.pone.0287318 (PMC10358888; doi:10.1371/journal.pone.0287318)
Supplement: S3 Table — (DOCX) [file pone.0287318.s004.docx]

**S3 Table. Excluded studies after full-text review**

| **Authors & Year** | **Reasons for exclusion** |
| --- | --- |
| Sanna et al., 2018^1^ | Not RCT |
| Cuadrado et al., 2020^2^ | Not RCT |
| Toni et al., 2016^3^ | Not RCT |
| Musat et al., 2018^4^ | Not RCT |
| Kitsiou et al., 2020^5^ | Not RCT |
| Israel et al., 2017^6^ | Not RCT |
| Yushan et al., 2019^7^ | Not RCT |
| Richards et al., 2015^8^ | Not RCT |
| Bettin et al., 2019^9^ | Not RCT |
| Nikolaeva et al., 2020^10^ | Not RCT |
| Rodriguez et al., 2018^11^ | Abstract Only |
| Sethi et al., 2019^12^ | Abstract Only |
| Ratajczak et al., 2019^13^ | Abstract Only |
| Passman et al., 2017^14^ | Abstract Only |
| Kitsiou et al., 2016^15^ | Abstract Only |
| De Lera et al., 2019^16^ | Abstract Only |
| Chousou et al., 2017^17^ | Abstract Only |
| Brachmann et al., 2014^18^ | Abstract Only |
| Singer et al., 2021^19^ | Not stroke as outcome |
| Poli et al., 2016^20^ | Not stroke as outcome |
| Petrovicova et al., 2015^21^ | Not stroke as outcome |
| Brachmann et al., 2016^22^ | Not stroke as outcome |
| Reiffel et al., 2017^23^ | Enrolled individuals were uncertain |
| Diederichsen et al., 2017^24^ | Enrolled individuals were uncertain |
