# Supplementary figures and images for: Effect of implantable cardiac monitors on preventing stroke: A systematic review and meta-analysis of randomized clinical trials
Source: PLoS One. 2023 Jul 20;18(7):e0287318. doi: 10.1371/journal.pone.0287318 (PMC10358888; doi:10.1371/journal.pone.0287318)

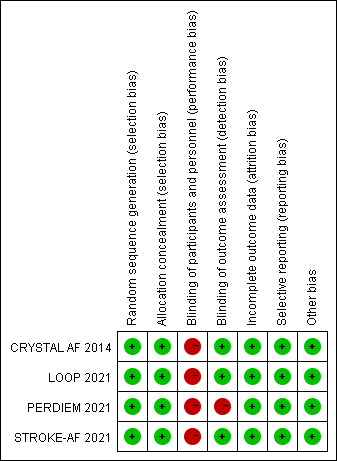


**S1 Fig. Risk of bias summary**

Supplement: S1 Fig — (DOCX) [file pone.0287318.s005.docx]

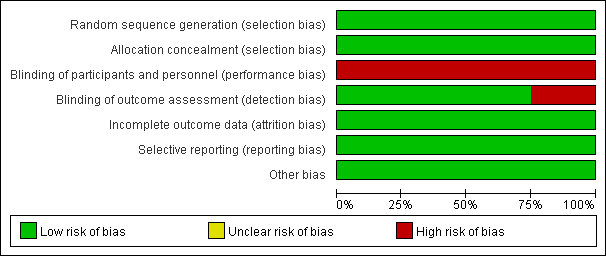


**S2 Fig. Risk of bias graph**

Supplement: S2 Fig — (DOCX) [file pone.0287318.s006.docx]

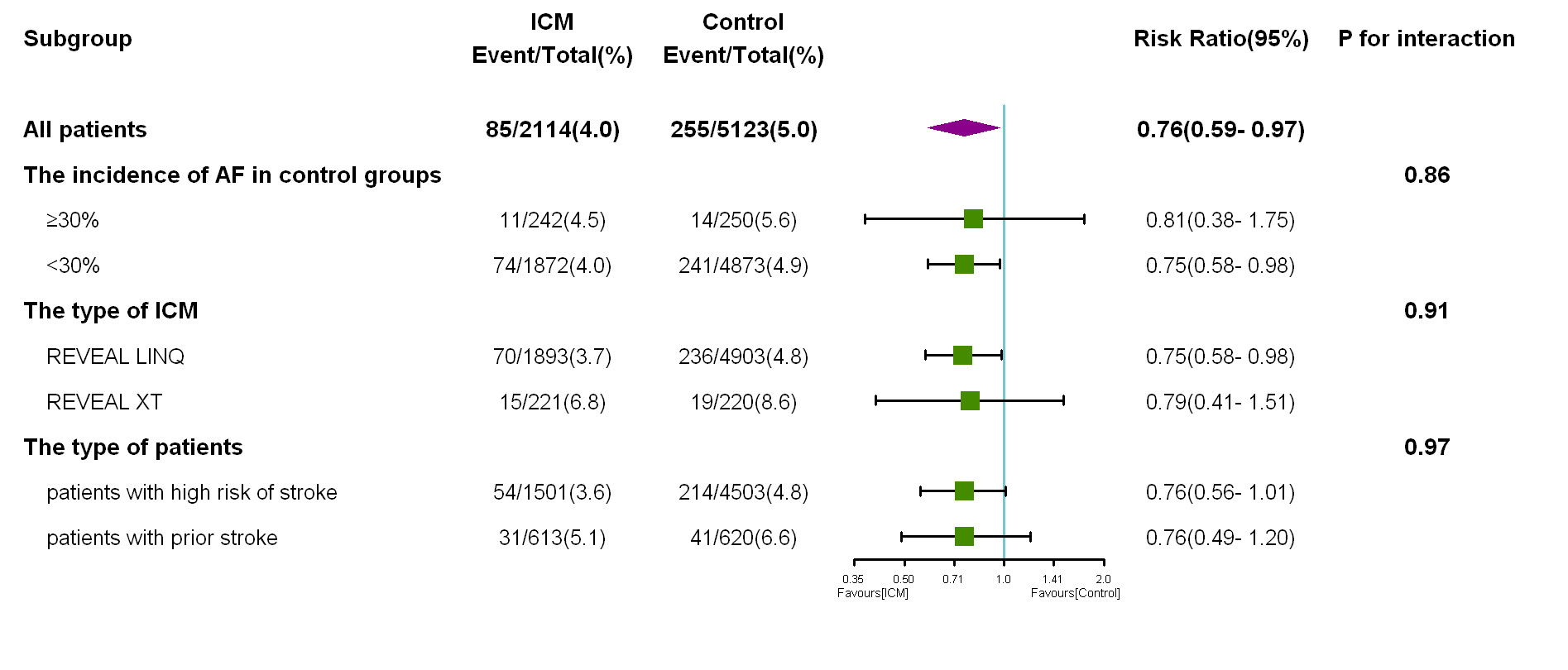
 **S3 Fig. Subgroup analysis of meta-analysis**

Supplement: S3 Fig — (DOCX) [file pone.0287318.s007.docx]
